# Nurture might be nature: cautionary tales and proposed solutions

**DOI:** 10.1038/s41539-020-00079-z

**Published:** 2021-01-08

**Authors:** Sara A. Hart, Callie Little, Elsje van Bergen

**Affiliations:** 1 Department of Psychology, Florida State University, Tallahassee, FL USA; 2 Florida Center for Reading Research, Florida State University, Tallahassee, FL USA; 3 Department of Psychology, University of New England, Armidale, NSW Australia; 4 Department of Biological Psychology, Vrije Universiteit Amsterdam, Amsterdam, The Netherlands

**Keywords:** Human behaviour, Education

## Abstract

Across a wide range of studies, researchers often conclude that the home environment and children’s outcomes are causally linked. In contrast, behavioral genetic studies show that parents influence their children by providing them with both environment and genes, meaning the environment that parents provide should not be considered in the absence of genetic influences, because that can lead to erroneous conclusions on causation. This article seeks to provide behavioral scientists with a synopsis of numerous methods to estimate the direct effect of the environment, controlling for the potential of genetic confounding. Ideally, using genetically sensitive designs can fully disentangle this genetic confound, but these require specialized samples. In the near future, researchers will likely have access to measured DNA variants (summarized in a polygenic scores), which could serve as a partial genetic control, but that is currently not an option that is ideal or widely available. We also propose a work around for when genetically sensitive data are not readily available: the Familial Control Method. In this method, one measures the same trait in the parents as the child, and the parents’ trait is then used as a covariate (e.g., a genetic proxy). When these options are all not possible, we plead with our colleagues to clearly mention genetic confound as a limitation, and to be cautious with any environmental causal statements which could lead to unnecessary parent blaming.

Most parents spend hours fretting over decisions about the environment they provide to their children. The scientific literature mirrors this idea. Across a wide range of studies from many psychological domains, researchers often conclude that the environment parents provide and children’s outcomes are causally linked, through environmental transmission (see Box 1). For example, a study examining the association of having a home library as an adolescent and later adult literacy, numeracy and technology skills drew our attention because of in-depth coverage in the Guardian (https://www.theguardian.com/books/2018/oct/10/growing-up-in-a-house-full-of-books-is-major-boost-to-literacy-and-numeracy-study-finds). This study used a very rich and well-powered dataset, and found a correlation between the number of books in adolescents’ homes and literacy performance in adulthood. They conclude that “growing up with home libraries boosts adult skills”, inferring a causal connection^1^. This is depicted in Fig. 1. Here we discuss how the correlation between the environments parents provide, the “rearing environment”, and their children’s outcomes can indeed be fully due to a causal association, or importantly, can also be partly or fully due to a genetic confounding, illustrated in Fig. 2 (see Footnote 1 in the Supplementary Notes). After highlighting the problem, we suggest ways that psychological scientists can examine research questions related to the rearing environment and children’s outcomes in ways that account for, or at least acknowledge, genetic confounding.

**Fig. 1:**
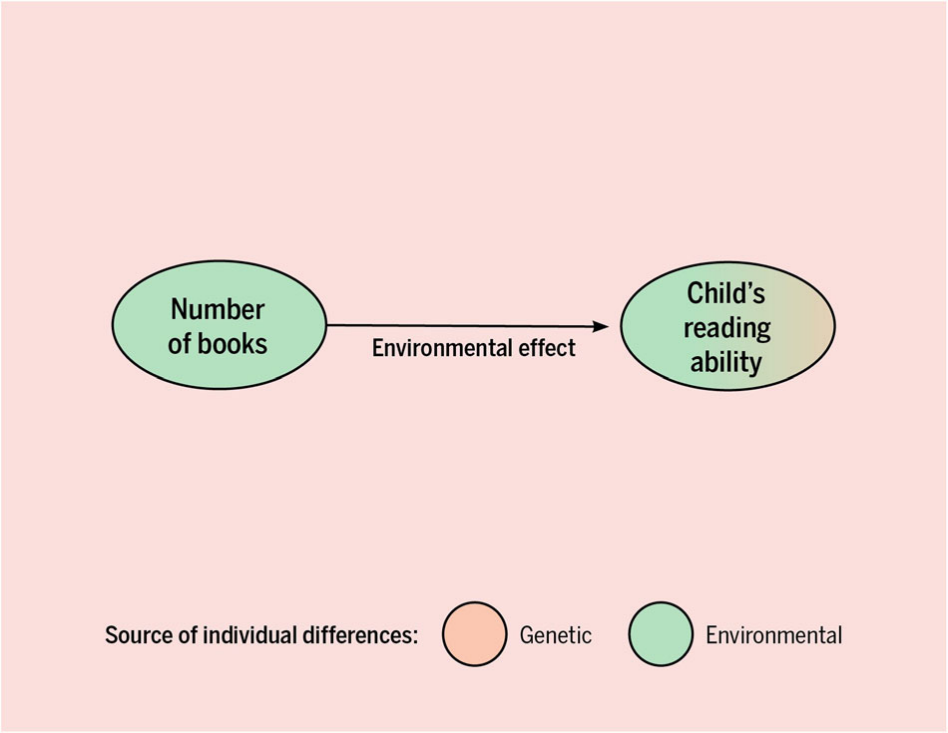
An example of a direct environmental transmission effect. Number of books in the home is thought to be an environmental causal effect on children’s reading ability. Figure by ref. ^66^ available at https://bit.ly/3gl8MVk under a CC BY 4.0 license.

**Fig. 2:**
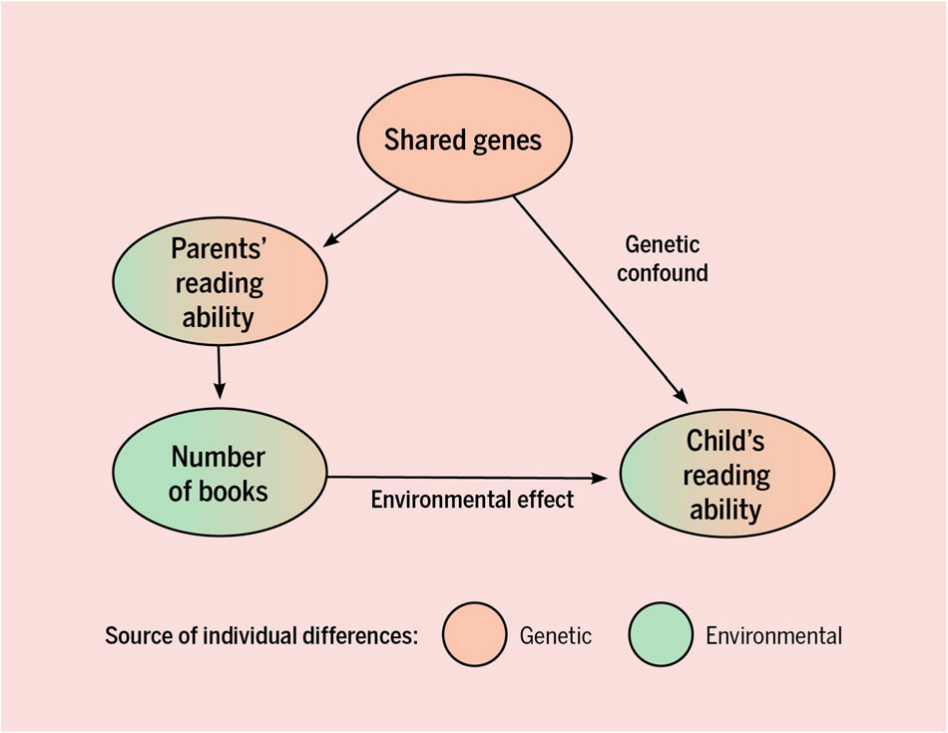
An example of how genetic confounding works (note, only one parent drawn, for simplicity). Parents share genes related to reading ability with their children, and also control the number of books in their home. This creates gene–environment interplay. It is important to note that the environmental effect may still have a causal role, even with gene–environment interplay. If genes play a role but are not modeled (as in Fig. 1), the correlation between the environmental measure and the child’s trait is genetically confounded. Here, the role of genes is modeled, allowing for an estimation of the genetic effect and the environmental effect. Figure by ref. ^66^ available at https://bit.ly/31c52z9 under a CC BY 4.0 license.

## Genetic control of exposure to the environment

Decades of work from behavioral genetics show that children’s traits are influenced by both genetic and environmental effects^2,3^. Likely more surprising to hear for most is that genetic influences are often seen on measures of the “environment”, suggesting that the contexts surrounding children are partly under genetic control^4^. For example, a meta-analysis found cumulative support for genetic influences on the parenting children received^5^. This idea, that there is genetic influence on exposure to environments, is called a gene–environment correlation. A gene–environment correlation describes the process by which a person’s genotype influences their exposure to the environment^6^. It is certainly not the case that genes are doing this directly, but instead genotypes matter for aspects of our personality, behaviors and cognitions, which then influence how we interact with our environment and how others interact with us^7^. This concept of an individual purposely and dynamically interacting with their surrounding environment is not limited to behavioral genetics; similar processes have been described in other literatures, for example person-centered interactions^8^ and the Selective Optimization with Compensation^9^.

Specifically, there are three types of gene–environment correlations that can result in genetic confounds^6^. First, a “passive gene–environment correlation” describes the association between the genotype a child inherits from their parents and the environment the child is raised in. Another way to think of it is that genes are a third variable which influence both the rearing environment a child receives as well as the child’s own traits, via genetic transmission from parents to child. This means it is not possible to draw causal conclusions between the rearing environment and children’s traits. For example, home environments have been found to be less chaotic for children with high effortful control, with results indicating that the same genes in parents which contribute to the levels of structure in their home (i.e., factors such as absence of noise and crowding, as well as presence of structure and routine) are also transmitted to their children and contribute to effortful control^10^. Second, an “evocative gene–environment correlation” is when a person’s genetically influenced trait elicits, or evokes, a specific response from others in the environment. For example, it has been found that a person’s genes are associated with being rated as “more likeable” by others, meaning how others perceive you as a social partner, and then likely interact with you, is influenced by your genes^11^. Third, an “active gene–environment correlation” describes the association of a person’s genetically influenced traits and the environments they select. For example, the genetically influenced personality trait of socialization, measured in childhood, was associated with exposure to risky environments related to substance abuse in adolescence, in that children with low socialization were exposed to more risky environments^12^. All three have the potential to cloud the true combination of genetic and environmental influences transmitted between parents and children (i.e., genetic confounding), but it is theorized that passive gene–environment correlations have a greater effect in childhood^13^, and as such passive gene–environment correlations are the focus of our review.

To give an example of how (passive) gene–environment correlations can result in genetic confounding in studies focused on the rearing environment, a high impact finding reported that parents with higher math anxiety have children with higher math anxiety, solely due to the home environment^14^. The authors attribute helping with math homework as the causal environmental factor, concluding that parents with high math anxiety should not help with their children’s math homework. This causal connection could exist, but equally parents with math anxiety also pass on genetic (and environmental) risks related to both lower math cognition and higher math anxiety^15^. Because this genetic transmission was not controlled for, causal claims and associated parenting advice are not justified.

Another example, this time from the medical literature, examined the intergenerational transmission of smoking behavior from parents to adolescents, concluding that “the attitudes, beliefs, and behaviors toward [adolescent] cigarette use are learned through [parent] modeling”^16^. Again, this study focused on the environmental transmission from parents to offspring without accounting for the transmission of genes related to smoking behaviors and risk-taking behaviors^17^. Furthermore, the authors conclude that smoking cessation interventions in adults can reduce smoking in subsequent generations. Parent-centered interventions might help to reduce adolescent smoking, but what is overlooked is that children carry their own genetic risks for smoking, and direct intervention with the adolescents^18^ could more strongly influence their smoking behaviors.

We are certainly not the first to point out this familial transmission confound within the ecological literature. Indeed, nearly 40 years ago, Scarr and McCartney proposed that “the human experience and its effects on development depend primarily on the evolved nature of the human genome”^13^, and nearly 30 years ago Plomin and Bergeman^19^ addressed the prevalence of genetic confounding by illustrating that genetic influences are found on most if not all environmental measures. Since then, several reviews have pointed to multiple examples from parental warmth to alcohol use to depression where causal pathways from parent behavior to child outcomes are reported, without accounting for genetic confounding^20–22^. These reviews have called for researchers to use caution with causal statements, and to address genetic confounding in their limitations. Further, they have asked for journal editors and reviewers to be better watch-dogs in this endeavor; to insist that manuscripts adhere to these standards. However, based on our experience listening to conference presentations and reading press releases and newspaper articles, we believe these guidelines are not yet being met.

We believe a reason why these previous reviews have not successfully changed minds and methods is because they have not given actionable correlational design solutions to researchers outside of behavior genetics. Therefore, when faced with not doing the work or publishing work with only a potential genetic confound, researchers have chosen the latter. Therefore, in the following section we will give many possible solutions, from genetically sensitive designs to design solutions that work in lieu of genetically sensitive data, and finally, a renewed call for changes in reporting standards.

Box 1 A glossary of some key terms

**Table:** 

Environmental transmission (also called: cultural or phenotypic transmission):	Transmission of traits from parents to their children by non-genetic means. It is used to describe when parents’ traits impact their child’s traits through the environment they create.
Familial Control Method:	Using a measure of the same trait in the parent as the child as a covariate in models estimating the effect of the rearing environment. That covariate then serves as a proxy control for the genetic transmission effect.
Familial transmission:	Transmission of traits from parents to their children, both by genetic and non-genetic means. Familial transmission gives rise to parent–child resemblance.
Gene–environment correlation:	Genetic influence on the exposure to the environment. There are three types: passive, evocative, and active (see text).
Genetic confounding:	Confounding due to gene–environment correlation. Here we focus on confounding due to a *passive* gene–environmental correlation, describing a situation where the influence of parental traits on children’s traits is not (solely) due to environmental transmission.
Genetic transmission:	Transmission of traits from parents to their children by genetic means (i.e., children inherit genes from their parents for a given trait).
Genotype:	An individual’s complete heritable information. A combination of alleles for a specific gene or across the whole genome.
GWAS:	Genome-wide association study. Identifies genetic variants (i.e., SNPs) across the genome that are linked to a trait.
Phenotype:	An individual’s observable traits, like eye color, reading ability, or parenting style.
SNP (pronounced “snip”):	Single nucleotide polymorphisms (SNPs). A single position in a DNA sequence that varies among individuals. For example, if a particular SNP can be nucleotide G or nucleotide C, then individuals can have GG, GC, or CC (one nucleotide from each parent). SNPs are the basis for genome-wide association studies.

## What researchers can do

The designs that we discuss below present a not all-encompassing but global overview of genetically sensitive designs and polygenic-scores (PGS) designs, and include a genetic-proxy control design (the “Familial Control Method”), which we recommend when genetically sensitive data are not available, as well as several other proxy control designs. These designs vary in how well they disentangle the genetic confound and in how challenging they are in terms of obtaining and analyzing the data.

### Genetically sensitive designs

**Fig. 3:**
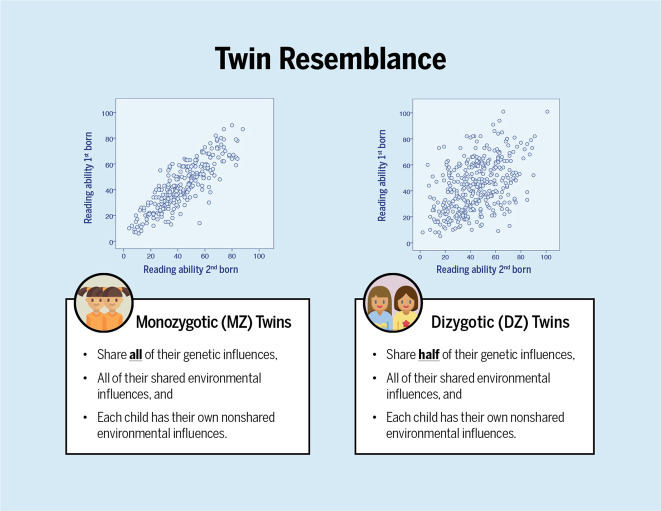
The logic behind twin research. The scatter plots depict how much the two types of (reared-together) twins resemble their co-twin on reading ability. Each dot represents the reading scores of both children within a pair. It can be seen that monozygotic twins are much more alike. From this, it can be concluded that differences between children are largely due to genetic differences. The data come from van Bergen et al.^34^ and represent word-reading fluency test scores in Grade 2 of twin pairs with complete data. The score is the number of words read correctly within 1 min. In this sample, the monozygotic and dizygotic twin correlations were 0.84 and 0.46, respectively, which yield estimates using the Falconer formulas^67^ of *A* = 0.76, *C* = 0.08, and *E* = 0.16 (see Fig. 4). Figure by ref. ^66^ available at https://bit.ly/3k4w2Ji under a CC BY 4.0 license.

**Fig. 4:**
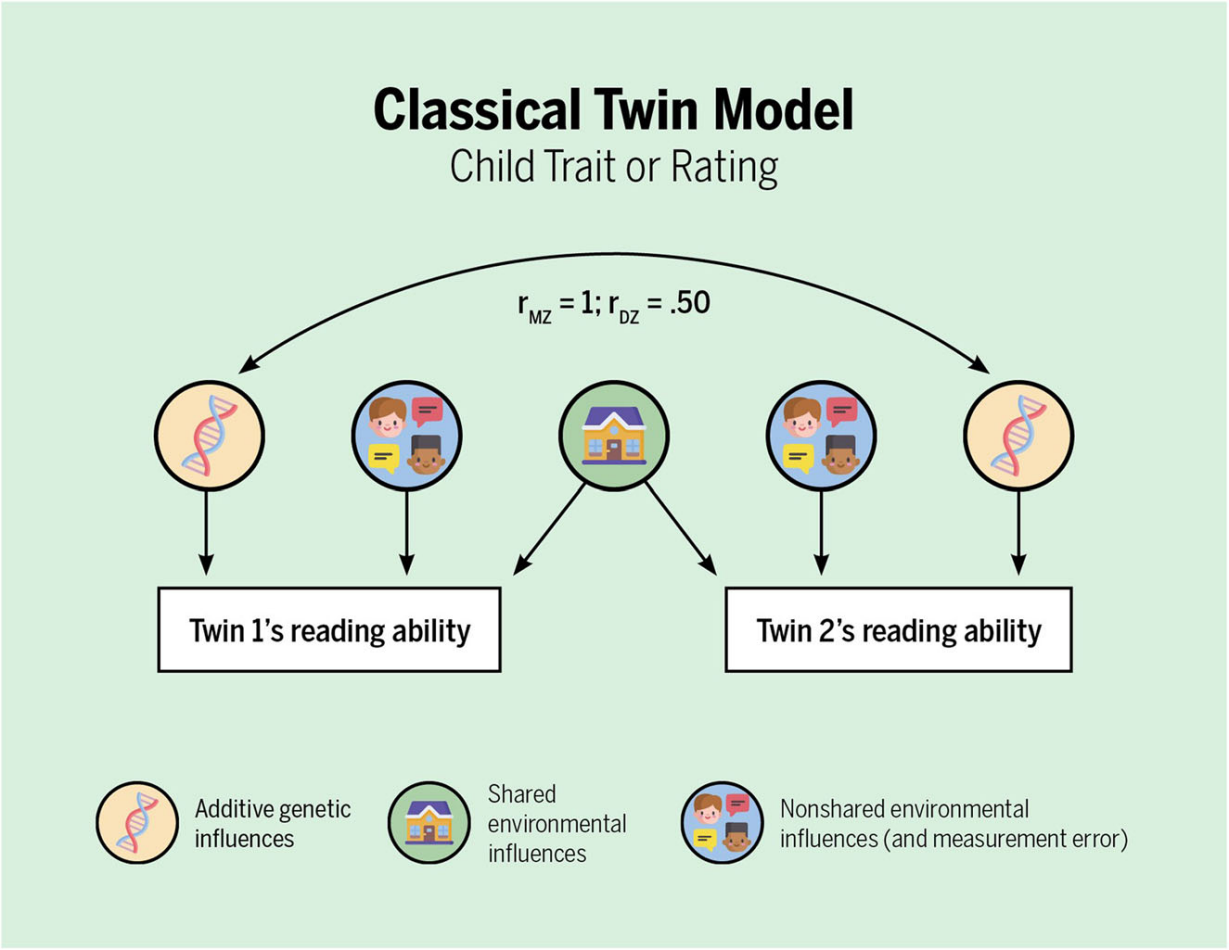
Simplified representation of the classical twin model. In behavioral-genetic models, the three sources of influences on individual differences are commonly labeled by the letters A, C, and E, respectively, stemming from **A**dditive genetic influences (also known as heritability, and sometimes represented by an *h*^2^ instead on an A), **C**ommon environmental influences (also known as shared environmental influences), and non-shared **E**nvironmental influences (and measurement **E**rror). Note that the latter are by definition uncorrelated between twins. See for a detailed representation of the classical twin model, for example, Figure A.9 in ref. ^23^; *r*_MZ_ = monozygotic twin correlation; *r*_DZ_ = dizygotic twin correlation. Figure by ref. ^66^ available at https://bit.ly/2Xkr29P under a CC BY 4.0 license.

Genetically sensitive designs are ideal for studying genetic and environmental influences and their interplay. These designs take advantage of samples of related individuals that differ in genetic relatedness (e.g., monozygotic and dizygotic twins; Fig. 3) or differ in environmental exposure (e.g., monozygotic twins reared apart). By far the most commonly used genetically sensitive design is the classical twin design. This design works because twins share either all (identical or monozygotic twins) or half (non-identical or dizygotic twins) of their genes^23^. Both types of twins share some parts of their environment such as their home, school, and neighborhood (referred to as common or shared environmental influences), and experience some aspects of their environments separately from each other such as peer groups, hobbies, or illness (referred to as unique or non-shared environmental influences). By comparing the average correlation between the two twins in a twin pair on a trait for monozygotic versus dizygotic twins, variance can be partitioned into additive genetic influences or heritability, shared environmental influences, and non-shared environmental influences (see Footnote 2 in the Supplementary Notes) (Fig. 4). Heritability of a trait is indicated if the correlation between monozygotic twins is higher than that of dizygotic twins. Shared environmental influences are estimated by subtracting the heritability estimate from the monozygotic twin correlation, and the estimated shared environmental influences is larger when the correlation coefficient between monozygotic versus dizygotic twins are close in magnitude. Finally, non-shared environmental influences are estimated by subtracting the monozygotic twin correlation from one.

With regard to disentangling possible genetic confounds and instead studying the direct effect of specific aspects of the rearing environment, classical twin studies are limited because both type of twins commonly share their rearing environments. For example, twin children growing up together are exposed to the same home library or household income, so monozygotic and dizygotic twin resemblance cannot be compared for these types of environmental measures. However, a classical twin study can begin to separate the direct effect of the home environment in two cases. First, child twins can be asked to individually rate their own rearing environment (Fig. 4). Since individual experiences are correlated with genetic predisposition, monozygotic twins often rate their experiences of the home environment more similarly with each other than dizygotic twins do. Therefore, when child twins can report their own ratings of their rearing environment, these estimates can serve to differentiate monozygotic and dizygotic twins. Twins who are children might not differ in how many books they have in the home, but they will likely differ in how much their parents read to them, or how much their parents monitor their reading. In these cases, the extent to which aspects of children’s rating of their rearing environment do not show entirely environmental influences, in other words, some heritability is measured on the “rearing environment”, this infers that there is a genetic confound, via a passive gene–environment correlation^4,19^. Using child twin ratings of their rearing environment, Hanscombe et al.^24^ found that 22% of the variance of chaos in the home was attributable to genetic factors, and moreover, 37% of association between chaos in the home and school achievement was due to shared genes. This suggests that this “environmental” variable of chaos in the home, measuring noise and lack of structure in the home, is partially genetically confounded. This means that chaos in the home does not have a completely direct, or causal role, on children’s school achievement.

The second way that the classical twin model can be used to identify the direct effect of the home environment, free of genetic confounding, is by focusing on the environment that adult twins create (see Fig. 4, but replace “reading ability” with “books in their home” or the like). When twins are adults they can differ in how many books they own and the income of their household, so genetic and environmental influences on their home environments can be studied. These studies quantify genetic and environmental influences on the home environment that twins create^4^, but they do not quantify the influence of these home characteristics on outcomes in their offspring.

Other genetically sensitive designs that can address the direct effect of the rearing environment, after accounting for genetic confounding, are adoption studies, within-family sibling studies, and twin-family studies^25^. In an adoption design, resemblance between adopted children and their biological parents is due to heritability (plus the prenatal environment). In contrast, resemblance between adopted children and their adoptive parents is fully due to the environment that the parents have provided. Another way to examine the rearing environment while partially controlling for genetic confounding is to use non-twin biological siblings within a family^26,27^. Because biological siblings, like dizygotic twins, share half of their genes, sibling resemblance on a trait suggests the influence of genetic factors along with some shared environmental influences. However, non-twin siblings can differ on several aspects of the rearing environment such as family size or parental health and age at birth, therefore, any dissimilarity between siblings can help to determine the influences of these non-shared aspects of the rearing environments on a given trait. Sibling designs have also recently incorporated the use of genome-wide PGS which strengthen their ability to control for confounding by disentangling direct genetic influences from gene–environment correlations^28,29^. PGS designs are discussed in more detail, below.

**Fig. 5:**
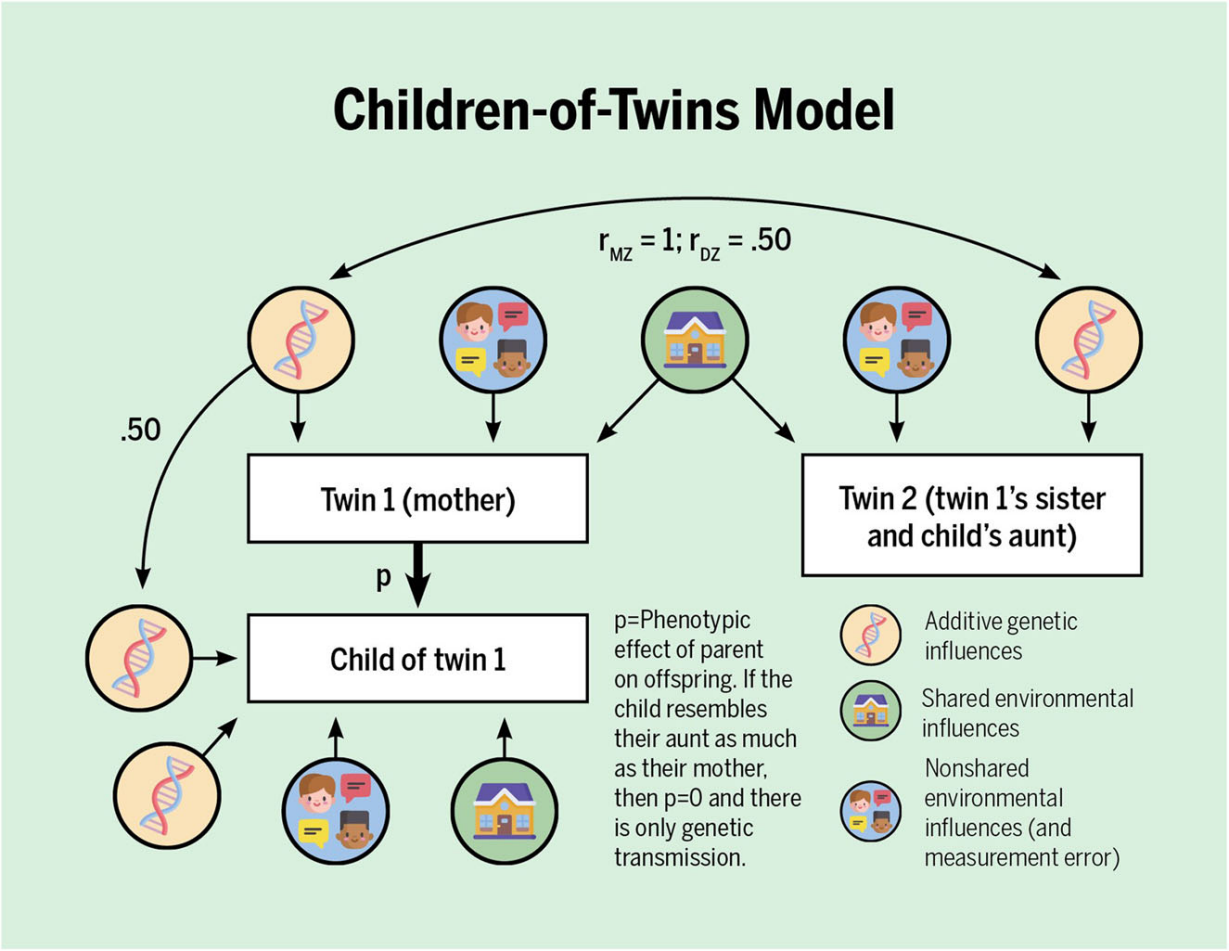
Simplified representation of the children-of-twins model. In the given example, the (adult) twins are sisters. The genetic transmission (left hand side) is fixed at 0.50 because parents and children share 50% of their genome. The other set of genes that influence the child trait (bottom left) are genetic influences that explain variance in the child trait but not the parent trait. The crucial test for presence of environmental transmission is whether the p-path is significant. Note that ‘child’ can refer to child or adult offspring. See, for the full and detailed model, ref. ^31^. Figure by ref. ^66^ available at https://bit.ly/2D0aNYJ under a CC BY 4.0 license.

Twin-family studies include twins and their family members, like young twins and their parents, or adult twins and their children. The latter, referred to as children-of-twins design (Fig. 5), is particularly suitable to study the effect of children’s rearing environment, free of genetic confounding^21^. Put simply, consider a mother who has an identical twin sister. The mother’s son shares half of his genetic variants with his mother, but also with his aunt. If for a given trait he resembles his mother as much as his aunt, this suggests that the resemblance is fully due to shared genes. Conversely, if he is more like his mother than aunt, this demonstrates that the resemblance between mother and son is at least partially due to the environment provided by his mother (see Footnote 3 in the Supplementary Notes). There are even more complex extended twin family designs, described well in Keller et al.^30^ and McAdams et al.^31^.

In sum, genetically sensitive designs can assess whether the rearing environment is influencing children’s outcomes, outside of genetic confounds. Although they are observational and hence cannot establish causality, or the absence thereof, they can strongly infer causality above and beyond the majority of typical observational studies. An important point to make is if a genetically sensitive study suggests no direct causality of the rearing environment on children’s outcomes, it does not imply that intervening is pointless. Successful parenting interventions are able to experimentally induce changes in parents’ skills or behaviors, which then causally improve child outcomes. Thus, observational studies, such as all the genetically sensitive designs described here, and experimental studies (preferably randomized controlled trials^32^) answer related but different questions: the first on “what is”, so causality in the natural situation, and the second on “what could be”, so causality due to intervening^33,34^.

Returning to genetically sensitive designs, the disadvantage is that they require access to such data, which are challenging to collect and analyze. We note for a reader interested in using twin data to better answer their questions about the direct role of the rearing environment, twin datasets are increasingly becoming publically available. For example, TwinLife (https://www.twin-life.de/en), TEDS (http://www.teds.ac.uk/researchers), NLSY kinship links (http://nlsy-links.github.io/NlsyLinks/), Netherlands Twin Register (http://tweelingenregister.vu.nl/research), and others are available online or via application. In addition, there is a data sharing culture in the behavioral genetics community, and most will likely share when asked. We suggest that researchers consider using these resources to better test their research questions.

### PGS designs

**Fig. 6:**
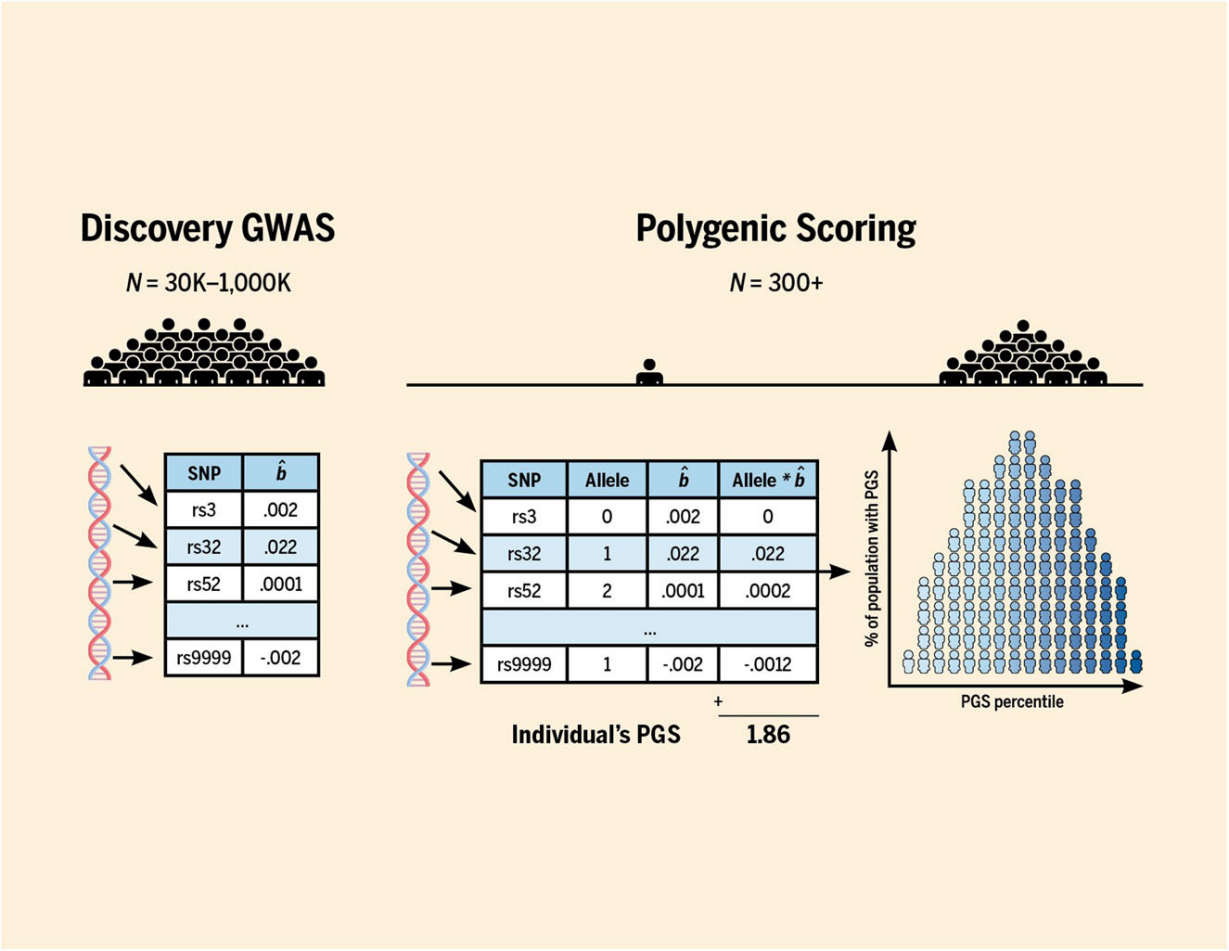
A polygenic score (PGS) indexes an individual’s genetic predisposition for a cerain trait or disease (see also^68^). Left panel: A published genome-wide association study (GWAS) serves as an external database. In an extremely large sample, a GWAS estimates tiny associations (${\hat{\mathrm b}}$) between the trait of interest and millions of genetic variants. Specifically, the genetic variants studied are single-nucleotide polymorphisms (SNPs), located across the genome. Middle panel: Polygenic scoring can be done in a sample that was not part of the GWAS. For each individual in this sample, the SNP effects (${\hat{\mathrm b}}$) are multiplied by the number of trait-associated alleles (0, 1, or 2) the person carries. These values are summed across all SNPs to arrive at the individual’s PGS. Right panel: The resulting PGSs across individuals in that sample are normally distributed. If the trait of interest is a disorder, like ADHD, the individuals in the right tail have the highest genetic risk for developing ADHD. PGSs are not yet strong enough for predictions at the individual level, but see the main text for examples of how PGSs advance science at the group level. Figure adapted from ref. ^69^. Figure by ref. ^66^ available at https://bit.ly/2BPcCXP under a CC BY 4.0 license.

A new avenue to study intertwined genetic and environmental effects employs genome-wide PGS. This method relies on genome-wide association studies (GWASs) which pinpoint genetic variants (i.e., single nucleotide polymorphisms (SNPs)) that are linked to a trait (Fig. 6). The most powerful GWAS to date (*N* > 1 million) has identified 1271 genetic variants associated with educational attainment^35^. Each of them has a tiny effect, but these tiny effects can be summed in a PGS. The PGS, calculated for all (unrelated) individuals in an independent sample, explains 12% of the variance in educational attainment. Note that twin studies estimate the heritability of educational attainment at 40%^36^, so the PGS currently captures less than one-third of this; the remainder is the “missing heritability”^37^.

**Fig. 7:**
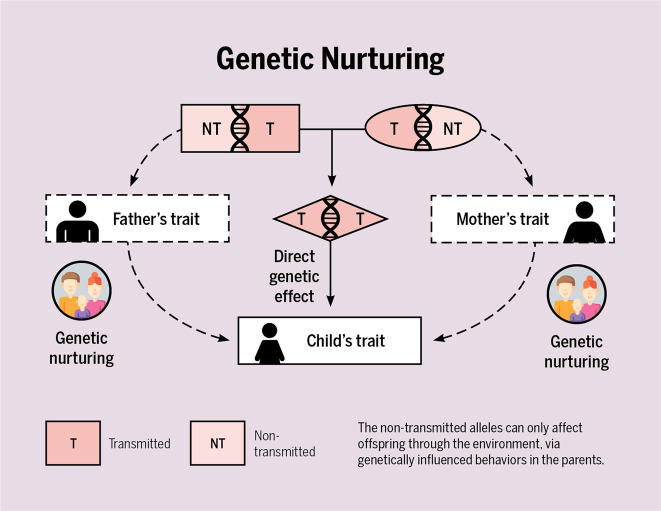
Simplified representation of the genetic nurturing design. In this design, one needs genotypes of parents and offspring, and a measured trait in the offspring generation only. The trait in the parents, for example educational attainment, is unobserved and indexed by a polygenic score of, in this example, educational attainment. The child receives half of the genotypes of father (top left) and mother (top right) and these transmitted alleles influence the child trait directly. The parental alleles that the child does not receive can still influence the child trait indirectly, via genetically influenced behaviors in the parents (denoted by the dotted genetic-nurturing paths). Genetic nurturing is present if the polygenic score of the *untransmitted* alleles explains a significant proportion of the variance in the child trait. The proportion of variance explained by the polygenic score of the *transmitted* alleles include both genetic nurturing and direct effects. Note that ‘child’ can refer to child or adult offspring. T = transmitted, NT = non-transmitted. Figure adapted from ref. ^39^. Figure by ref. ^66^ available at https://bit.ly/2PjpkRu under a CC BY 4.0 license.

As we speak, novel methods are being designed to disentangle nature and nurture that draw on PGS. Below, we list some examples of recent developments. First, Dolan et al.^38^ bring PGS into the classical twin design. By doing so, one can estimate the gene–environment correlation, rather than assume it is absent. Second, Lee et al.^35^ and Selzam et al.^29^ found that for cognitive traits, the predictive power of a PGS within a family was about 50% lower than across unrelated individuals. The attenuation of the PGS’ predictive power within families suggests that passive gene–environment correlations (as captured by the PGS) contribute to children’s cognitive development. As a third example, both Kong et al.^39^ and Bates et al.^40^ separately proposed the same design incorporating parental and offspring PGS to disentangle environmental transmission from genetic transmission (i.e., account for the genetic confound between the home environment and child outcomes). In both cases, the researchers split the genetic variants of the parents in half—those that the parent had and had not transmitted to the offspring—and calculated for each half the PGS for educational attainment (Fig. 7). The researchers do this because of the biological fact that a parent only transmits a random half of their genes to their child. And for this design to work, both parents and their child must be included (but see ref. ^41^ for a work around). Amazingly, what the researchers found was both sets of parent PGS predicted adult offspring’s educational attainment. The predictive value of the transmitted PGS was unsurprising, as this captures directly transmitted genetic effects. But the predictive value of the non-transmitted PGS was not certain. If non-transmitted PGS influence children’s traits, this effect must be environmental, likely acting through rearing behaviors that affect the child’s development. Kong et al.^39^ aptly coined this genetic effect through the rearing environment “genetic nurturing”. Belsky et al.^42^ did a similar analysis but with an updated PGS score. Interestingly, when this design is expanded to include grandparents, there is little evidence for genetic nurturing from the grandparent generation^43^. Fourth, Wertz et al.^44^ incorporated both PGS of mothers and children, as well as direct measures of parenting. They showed that mothers’ cognitive stimulation explained the relation of the maternal non-transmitted PGS to child educational attainment. This indicated that there is a direct environmental transmission of parenting on children’s outcomes, unconfounded by correlated genetic transmission. Finally, de Zeeuw et al.^45^ and Willoughby et al.^46^ both used the full genetic-nurturing design (employing DNA of children and both parents) and found (thereby replicated) genetic-nurturing effects on adults’ educational attainment. Crucially, for outcomes in childhood, academic achievement and ADHD-symptoms, Zeeuw et al.^45^ only found direct genetic effects; no genetic nurturing. They concluded that a large contributor to why the rearing environment predicts child outcomes may well be intergenerational transmission of genetic effects.

At the moment, measured genetic variants only explain small proportions of variance and the papers mentioned above may be seen by behavioral researchers as only a proof-of-principle. Nevertheless, these exciting developments will gain in strength when increasingly larger GWASs of all sorts of traits yield more refined PGS. By this we mean that PGSs will begin to explain more and more portions of the variance in outcomes we are interested in, with the hope that eventually they will reach the theoretical upper limit of SNP heritability. Even then, using them as a genetic control (i.e., as a covariate) will continue to underestimate the total genetic effects we are looking to control. PGSs account for only one type of genetic effect, namely common variants. There is increasing evidence that traits such as educational attainment are influenced by not only common variants, but also rare variants^47,48^. Another concern is that new work is indicting that a PGS is not a measure of only genetic variance. Instead, it likely represents not only causal genetic effects, but genetic ancestry, assortative mating, gene x environment interactions, direct environmental influences (i.e., genetic nurture), and environmental confounds from, for example, SES^49,50^. Therefore, a measure of genes (i.e., a PGS) can predict trait variance via environmental routes. This parallels our earlier notion that a measure of the environment can predict trait variance via genetic routes.

In summary, at the moment the PGS is not a perfect “genetic control”, as it does not account for all of the genetic effects and also accounts for other effects, including the very environment we are interested in. But, we believe that next to no control, using a PGS as a statistical control is still better. Costs of genotyping are falling and the number of cohorts with genotype data is growing^51^. We predict that in the not-so-far future, using simply and cheaply collected genotypic information will become a regular part of the behavioral researchers’ data collection protocol, especially as the predictive validity of the PGSs increases. This means that PGSs will allow researchers to partly control for genetic confounds in their models. We foresee that it will be easier to use PGSs than rely on genetically sensitive designs.

### Genetic-proxy control designs: the Familial Control Method

The designs discussed above are the current gold-standards. However, as these types of samples discussed above are not currently easy to collect and analyze, we propose here a useful work around^52,53^. Our colleagues can measure the same trait in both the parents as the child, and use the paternal and maternal traits as covariates. We advise to assess the traits in both parents (but acknowledge the challenge that brings), because the child shares only 50% of their genes with one parent, but all of their genes with both parents. Hence, both parents are needed to best tag the child’s genetic liability. The parental traits, included as two covariates, then serve as a proxy for the familial transmission, including genetic transmission. In doing so, you have a proxy control for the familial effect. Hence, we term this method the Familial Control Method.

**Fig. 8:**
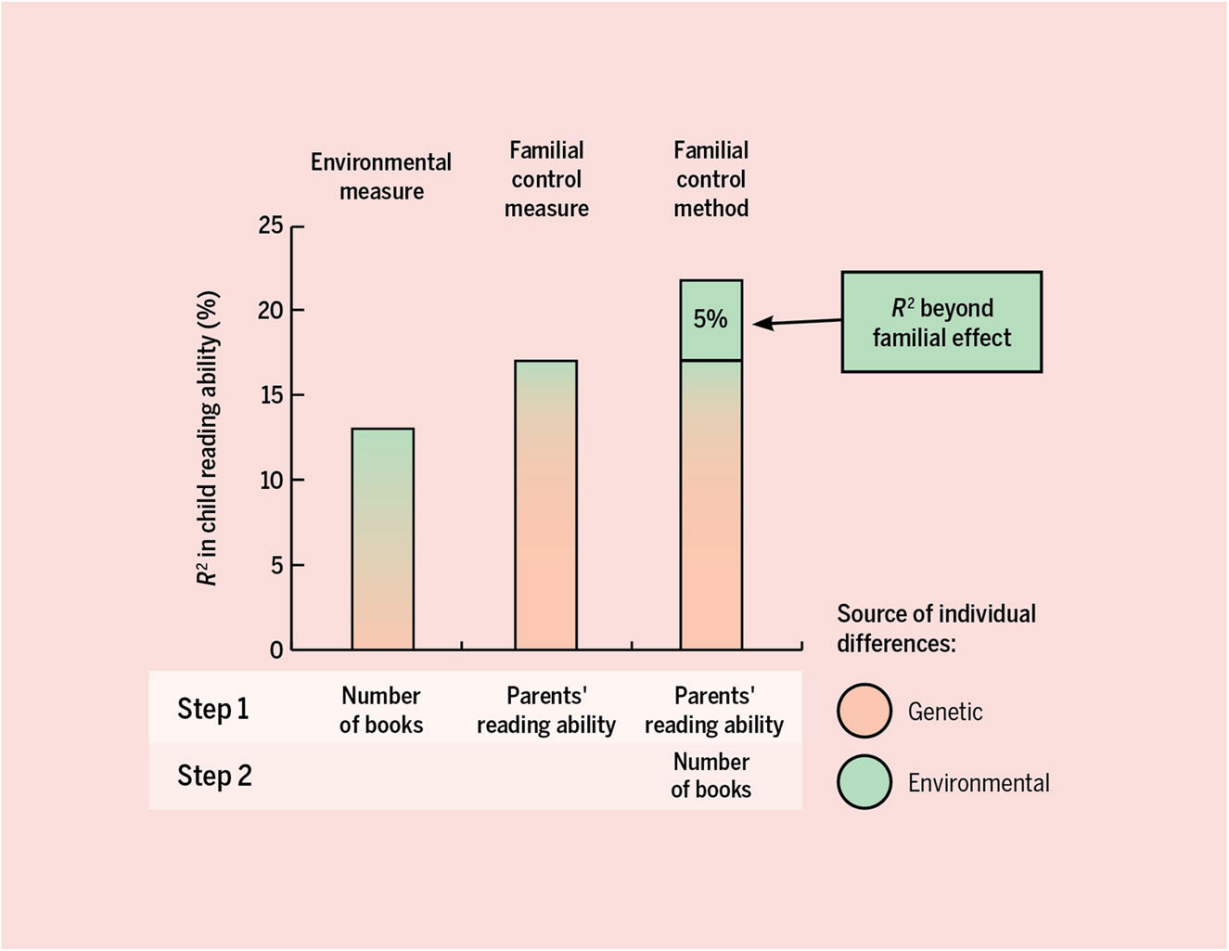
Visualization of the Familial Control Method, in which a child outcome is predicted in a step-wise regression, with in the first step the familial control measure (i.e., the trait in both parents) and in the second step the measure of the environment. The findings that are depicted here come from van Bergen et al.^53^. The key question is whether the environmental measure explains variance beyond the familial effect, as this indicates a genuine environmental effect. In the example given, this was 5% and significant. This was negligible and non-significant for the other environmental measures reported in ref. ^53^. Figure by ref. ^66^ available at https://bit.ly/2Pfjelh under a CC BY 4.0 license.

The Familial Control Method is designed for traits that are mostly transmitted from parent to child through genes rather than the environment, like reading ability^54,55^ (see Footnote 4 in the Supplementary Notes). Van Bergen et al.^53^ capitalized on this in studying whether children’s reading ability is influenced by the home literacy environment, like reading habits of the parents and the number of books in the home. Analyses consisted of straight-forward step-wise regression analyses, illustrated in Fig. 8. The home literacy environment correlated with children’s reading ability, but for most home-literacy indicators the effect was no longer significant after accounting for the reading ability of the parents. This suggests genetic confound rather than a genuine environmental effect. The one exception was the number of books children grow up with, which did explain variance over and beyond parents’ reading skills (Fig. 8). This suggests a genuine environmental effect on children’s reading by the number of books itself, or something related, like the value that the family places on reading^45^.

A similar approach was taken by Hart et al.^52^ in studying the effect of the home numeracy environment on children’s math ability. When a parent’s math ability was included in the model, some effects of aspects of the home numeracy environment on children’s math ability were attenuated, but most held up. A note of caution is that the skills of only one parent could be obtained and controlled for, so the study lacked a proxy for the genetic liability passed on by the other parent. The authors concluded that doing more math-related activities with your children does seem to directly boost their math.

We advise researchers who are interested in applying the Familial Control Method to search first in the literature for adoption and twin-family studies. Such studies with the outcome trait of interest (e.g., reading ability) assessed in both the parent and the offspring generation, test whether parent to offspring transmission is mainly genetic or environmental in nature^54,55^. However, such studies are scarce. If such studies for the trait of interest do not exist, a good starting point are classical twin studies. Traits with no or a small influence of the shared environment (referred to as C), like neurological traits, are more likely to be transmitted just genetically compared to traits with large shared-environment influences, like social values. Results of meta-analyses of twin studies on a very large number of traits can be found in Polderman et al.^2^ and the accompanying webtool (http://match.ctglab.nl/).

The Familial Control Method, using a parental trait as genetic proxy, is not watertight, and certain assumptions must be made for it to be effective for your research question (see Footnote 5 in the Supplementary Notes). First, if, for a certain trait, parent–child resemblance is not only due to genetic transmission but also environmental transmission, the Familial Control Method can be too conservative, as it also takes away some of the variance due to true environmental effects. However, one could argue that for many situations, being slightly too cautious in causal claims about environmental influences is less harmful than being too lax. However, this might not be the case for all researchers, and we encourage behavioral researchers to consider if being too conservative is actual harmful (e.g., for the effect of an unsafe home environment on child psychopathology) Second, as mentioned earlier, the trait measured in the parents should be the same or highly similar as the trait measured in the child. This means that traits which are not at least reasonably the same in childhood as adulthood (i.e., across birth cohorts and across the lifespan) would not work in this design. So the trait should be at least reasonably measurement invariant and relatedly, show reasonable genetic stability. Fortunately, for many phenotypes, children’s phenotypes are simply developmental precursors to the adult phenotypes (e.g., for reading ability^56^, and for ADHD^57^). A researcher must decide if the mentioned assumptions are appropriate for their trait of interest, but fortunately we do believe that these assumptions are reasonable for most to make. Third, correlations among the parent and child trait and the environmental measure of interest will be attenuated by measurement error. To reduce measurement error, one can do regressions according to the Familial Control Method in a structural equation modeling framework, with multiple indicators per construct. One can fit a model with as the outcome the latent child trait of interest, and as (correlated) predictors, the latent traits of both parents and the environmental measure of interest. If dropping the regression path ‘environmental measure → child outcome’ leads to a significantly worse model fit, this implies that the environmental measure is associated with the outcome above and beyond the familial effect. If the trait of interest is genetically transmitted, this equates to above and beyond genetic confounding, so suggests a direct environmental influence. The effect size here is given by the difference in explained variance in the child outcome of the models with and without the ‘environmental measure → child outcome’ path. Adopting a structural equation modeling framework with latent variables is especially advisable for constructs that are notoriously hard to measure reliably. Another advantage of this framework, compared to stepwise regressions, is that families with missing data can be retained.

It is likely the case that for most behavioral researchers interested in the direct role of the rearing environment, the Familial Control Method is currently the most feasible proxy genetic control. It does not require data of twin or adoption families, nor collecting DNA samples. In terms of prediction, parental traits capture more of the variance in children’s outcome than polygenic scores, so likely also capture more of the genetic confound. For the example of reading ability, it has been found that the abilities of both of the parents explain 21% of the ability of children^58^. In comparison, polygenic scores (based on the educational-attainment GWAS) have been found to explain only 2–5% of reading ability in children^59^, and more recently 5–14% of “educational achievement” (including reading, writing, speaking, listening, and mathematics) in ages 7 to 16 years^60^. Certainly this proportion of variance explained from simple polygenic scores is not trivial, and the predictive ability of polygenic scores is anticipated to increase in the coming years. However, for most behavioral scientists the trait in the parents is not only easier to measure, but currently also a better predictor. On a related note, the value of parental traits as predictors of child outcomes has been used for decades in studying precursors of developmental disorders. In such family-risk studies, children with a family history of say dyslexia, attention-deficit/hyperactivity disorder or autism are followed from an early age, before the disorder manifests itself. These children have an increased risk to develop the disorder^61,62^.

### Other proxy control designs

Other proxy controls such as sociodemographic factors (e.g., SES) have been used ubiquitously, but these statistical adjustments are not capable of accounting for genetic confounding as adequately as the Familial Control Method, for several reasons. First, although sociodemographic factors such as educational attainment have been significantly associated with genetic factors through twin studies (40%^36^) and GWAS (~14%^35^), the estimates are less than unity which indicates that genetic influences are not entirely responsible for individual differences in SES. Indeed, work examining the intergenerational transmission of SES has suggested that both genetic and environmental transmission occurs^63^. In this scenario where SES is transmitted through genetic and environmental pathways, when controlling for SES in data analyses, a proportion of variance attributable to other background or environmental factors is also being controlled for in the model, unintentionally leading to reduced associations between potentially important family-level predictors and child outcomes. In other words, you’d be throwing the baby out with the bathwater.

On the other hand, controlling for SES does not actually control for all of the genetic confounding. Say a researcher is interested in controlling for genetic confounding when examining the direct influence of books in the home on children’s reading. Parental SES is a proxy for parental reading skill, but not a perfect correlate (average correlation is 0.26^64^). Controlling for parental SES would not control for all of the potential genetic confounds on the association between books in the home and children’s reading ability. In conclusion, when controlling for SES, other potential sources of environmental variance are also being removed from the prospective models, and at the same time would not capture the extent of genetic confounding. We believe this would happen with other proxy control measures as well, outside of the Familial Control Method described above.

## Conclusion

**Fig. 9:**
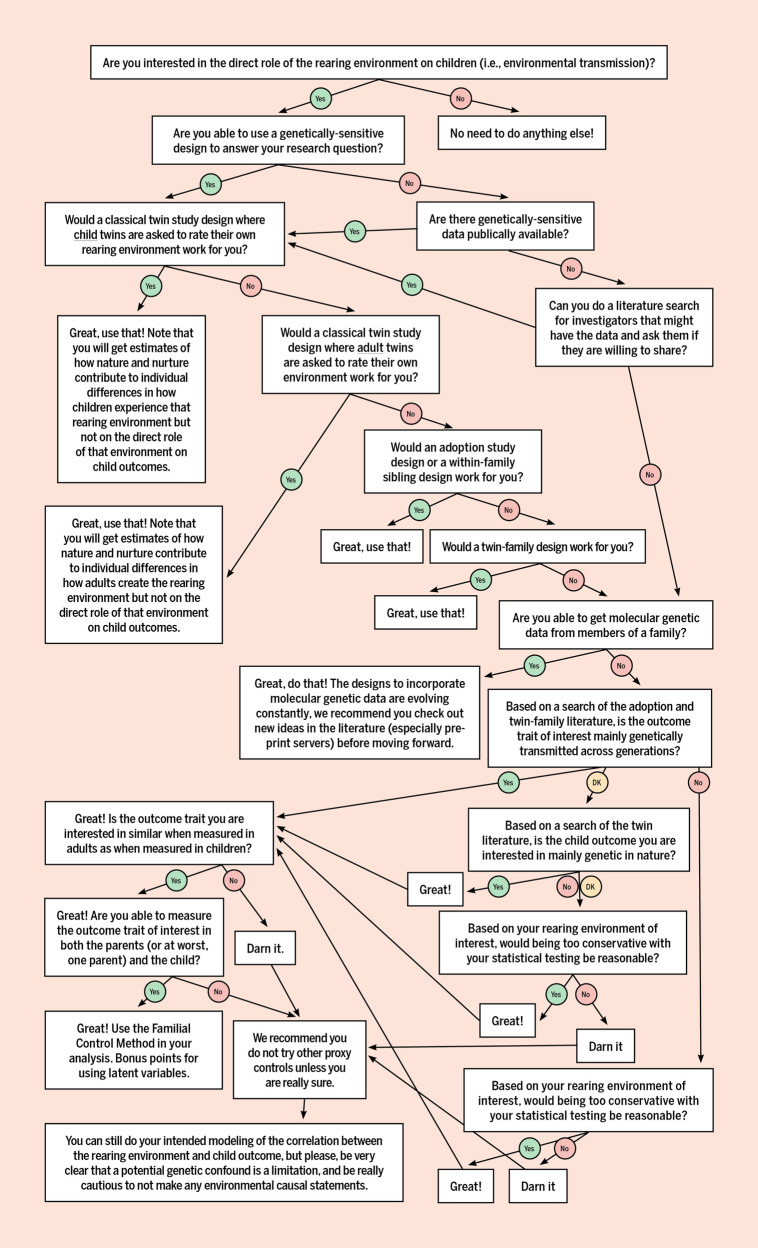
Decision flowchart for determining how to control for genetic confounding when examining the rearing environment. DK = don’t know. Figure by ref. ^66^ available at https://bit.ly/3gkM6Et under a CC BY 4.0 license.

Here we have laid out numerous ways that genetic confounding can be controlled for when examining the rearing environment, summarized in a decision flowchart (Fig. 9). We can certainly foresee times that none of these options are possible. Therefore, we conclude that in those instances, our colleagues need to clearly mention the possible genetic confounding as a limitation, and to be cautious with any environmental causal statements which could lead to unnecessary parent blaming or to interventions that are a waste of time and resources. To return to our first example, expecting all homes to have plenty of books is an idealistic goal, as it would surround all children with the opportunity to read if they wished. But unfortunately, having the opportunity to read as one wishes does not unlock the code of reading for all children. Reading is a skill that requires direct instruction and practice, and children with a family history of dyslexia themselves have a 45% chance of dyslexia despite adequate instruction and practice^61^. Simply having books around the home is not enough^65^, yet the message that parents are getting is that it is. The take home messages from that are that either parents who do not have the resources for a home library are hurting their children, or parents with children struggling to read are at blame because they did not have quite enough books in the home. This is unfair and inaccurate. In the end, we believe that it is important to discover true environmental effects as well as how genes and environments interplay, especially when malleable, because then we can focus as a field on creating and testing interventions that have a greater chance of directly improving children’s outcomes.

### Reporting summary

Further information on experimental design is available in the Nature Research Reporting Summary linked to this paper.

## Supplementary information

Related Manuscript File

Reporting summary

## Data Availability

Data sharing not applicable to this article as no datasets were generated or analyzed during the current study.

## Code availability

Code sharing not applicable to this article as no codes were generated during the current study.
