# Supplementary material for: Nurture might be nature: cautionary tales and proposed solutions
Source: NPJ Sci Learn. 2021 Jan 8;6:2. doi: 10.1038/s41539-020-00079-z (PMC7794571; doi:10.1038/s41539-020-00079-z)
Supplement: Supplementary file 1 — Related Manuscript File [file 41539_2020_79_MOESM1_ESM.pdf]

Supplementary Notes

Footnote 1. There is also likely environmental confounding, but that is not the focus here.

Footnote 2. As a note, these three sources of influence can also partially include gene-environment interplay when estimated using the classical twin design.

Footnote 3. To be precise, the p-path in Figure 5 is essentially the residual association between parent and child traits after accounting for modelled familial effects. It could be that this residual association comprises the effects of parents on children and/or the effect of children on parents. For parental traits like educational attainment the effect likely runs only from parent to child.

Footnote 4. We note for the reader more familiar with the behavioral genetics literature than the familial transmission literature that within individuals, a trait, like reading ability, is often influenced by both genetic and environmental effects (e.g., Little et al., 2017). However, when designs examine how a trait is transmitted from parents to children, often only genetic transmission is found. Environments can contribute to individual differences, but at the same time not impact why parents and children resemble each other.

Footnote 5. One of the reviewers agreed with most of our assumptions of the Familial Control Method, but strongly believed that these assumptions make the Familial Control Method not useful. We believe that this is actually an empirical question that we intend to follow up with.
